# Factors associated with treatment adherence to treatment among in patients with type 2 diabetes in Iran: A cross-sectional study

**DOI:** 10.3389/fpubh.2022.976888

**Published:** 2022-11-01

**Authors:** Nasrin Pourhabibi, Bahram Mohebbi, Roya Sadeghi, Elham Shakibazadeh, Mojgan Sanjari, Azar Tol, Mehdi Yaseri

**Affiliations:** ^1^School of Public Health Tehran, University of Medical Sciences, Tehran, Iran; ^2^Cardiovascular Intervention Research Center, Cardio-Oncology Research Center, Rajaie Cardiovascular Medical and Research Center, Iran University of Medical Sciences, Tehran, Iran; ^3^Department of Health Promotion and Education, School of Public Health, Tehran University of Medical Sciences, Tehran, Iran; ^4^Department of Internal Medicine, School of Medicine Endocrinology and Metabolism Research Center Afzalipour Hospital, Kerman University of Medical Sciences, Kerman, Iran; ^5^Department of Health Education and Health Promotion, School of Public Health, Tehran University of Medical Sciences, Tehran, Iran; ^6^Department of Epidemiology and Biostatistics, School of Public Health, Tehran University of Medical Sciences, Tehran, Iran

**Keywords:** adherence to treatment, health literacy, diabetes, type 2, HbA1c, patients

## Abstract

**Introduction:**

Diabetes is a chronic metabolic disorder that affects millions of people worldwide. Adherence to treatment is a key determinant to proper management. This study aimed to assess the factors associated treatment adherence in patients with type 2 diabetes.

**Materials and methods:**

We conducted this cross-sectional study on 704 patients with type 2 diabetes referred to three diabetes clinics in Kerman, Iran. We used treatment adherence questionnaire and functional communicative critical health literacy (FCCHL) to collect data and descriptive statistics, as well as Pearson correlation coefficient and multivariate regression analysis to analyze data. Significance level was <0.05.

**Results:**

The study results showed that health literacy, HbA1c, and income were main predictors of diabetes treatment adherence. The patients' adherence increased as their health literacy increased. The patients' HbA1c decreases as their adherence increased. We found a 2.54-point increase in the treatment adherence score for those with sufficient income and a 0.76-point increase in the treatment adherence score for those with relatively sufficient income compared with those with insufficient income.

**Conclusion:**

We found several factors affecting diabetes treatment adherence. Planning theory-based interventions can be helpful to improve the determinants.

## Introduction

Diabetes, a chronic metabolic disorder in the world, is becoming more common (1). Studies have reported a 10-point increase in the prevalence of diabetes by 2045 (2). The International Diabetes Federation has declared 10.6-point and 11.1-point increases in the prevalence of type 2 diabetes in Iran in 2030 and 2045, respectively. The disease mortality rate in Iranians under the age of 60 accounted for 39.7 percent of all deaths in 2019 (3). The disease management requires special self-care throughout the patient's life, including following a diabetic diet, physical activity, monitoring blood glucose, and adhering to medication regimen (4). These patients typically are reluctant to adhere to all of mentioned principles, so they will eventually require oral medications and even insulin therapy to control their blood glucose. One way to control diabetes is treatment adherence that improves blood glucose control and reduces glycosylated hemoglobin, resulting in fewer complications and all associated costs (5). Many patients with chronic diseases disregard the recommended medication regimen due to prolonged course of treatment and dissatisfaction with definitive treatment (6). Uncontrolled diabetes is frequently associated with physical and psychological complications, such as heart disease, stroke, hypertension, blindness, kidney failure, amputation, depression, and poor quality of life (7). Many therapists have been interested in how well patients with diabetes adhere to their treatment plans that is one of the most important challenges in controlling diabetes (8). Early discontinuation of medication, non-compliance with dietary instructions, and lack of physical activities are examples of patient-related factors, which influence treatment adherence (9).

A strong relationship exists between diabetes and overweight or obesity (10). Estimates suggest that ~90% of people with type 2 diabetes (T2D) have overweight or obesity (11). People with type 2 diabetes and high BMI have worse glycemic control (12). As BMI increases, achievement of target levels of glycated hemoglobin (HbA1c) declines (13); high BMI contributes to developing T2D-related complications, including neuropathy, nephropathy, cardiovascular disease, and peripheral vascular disease (14).

Some factors, like socio-economic status, have considerable effects on adherence to chronic drug regimens. Compatible with the comprehensive definition of WHO, health is influenced by the socio-economic, genetic, and biologic factors; social factors play an important role in patients' adherence to medical recommendations (15). The education level, occupation, income, housing, nutrition, environment, workplace, poverty, water, unemployment, stress, culture all have significant direct and indirect roles in the health status of individuals and communities (16). Some evidence suggests the effect of the socio-economic status on treatment adherence among patients with diabetes. For example, low level of socio-economic status can affect the outcomes of patients with diabetes, including disease mortality and complications (15).

Lack of adequate adherence to treatment regimens increase disease complications and healthcare costs, prolongs treatment duration, and double the mortality rate of these patients compared with other patients (17).

According to World Health Organization (WHO), diabetes self-management training facilitates the knowledge, skills, and ability necessary for diabetes self-care; health literacy affects patients' knowledge and awareness of their disease (7). Health literacy is an individual's capacity to acquire, process, and comprehend the basic information and services needed to make appropriate health decisions, including a set of reading, listening, analysis, decision-making skills in health situations (18). Health professionals classify health literacy into three main categories: the ability to read consent forms, drug labels and attachments, and other written information, the ability to understand written and oral information given by a physician, nurse, pharmacist, insurer, and the ability to follow pharmacological instructions and medical care (19). People with limited health literacy are less successful in managing diseases such as diabetes, indicating the importance of health literacy in self-care (20). Aseeri (21) demonstrated that health literacy could explain for 61% of self-care behaviors. People with greater health literacy engage in more self-care behaviors, which can improve the social support and quality of life of patients with diabetes (21). Hussain et al. (22) indicated that individuals with a higher level of health literacy were more likely to adhere to their treatment than those with a limited level of health literacy; people with adequate health literacy were less likely to alter their medication dosage without consulting a doctor than those with inadequate health literacy.

People's correct social, psychological, and health lifestyles affect their quality of life, so providing appropriate information for them and raising their health literacy can improve their social lives. Health literacy improves the lives of type 2 diabetes patients and enables them to take effective steps toward living a healthy life (23). This study aimed to determine the factors associated with treatment adherence in patients with type 2 diabetes in Iran.

## Materials and methods

### Study design and setting

We conducted this cross-sectional study in three diabetes clinics in Kerman in southeastern Iran (Erfan Salamat, Bahonar, and Shafa).

### Participants, sample size, and sampling

Patients over the age of 30, diagnosed with diabetes for at least six months, and with no confirmed psychological problems met the inclusion criteria. Exclusion criteria were pregnant women with gestational diabetes, type 1 diabetes, and underlying diseases not related to type 2 diabetes (such as various cancers and autoimmune disease, etc.). All eligible patients with type 2 diabetes were selected by convenience sampling method. Twenty-two thousand seven hundred were the total number of patients available in the three clinics. The sample size was estimated to be 585 using Cochran's formula for a definite population in accordance with the study's primary objective (*Z* = 1.96, *d* = 0.04). Regarding dropout probability, 750 questionnaires were distributed but 704 questionnaires were completed, with the response rate of 93.87%. Power analysis calculations with G^*^Power software (version 3.1.9.2; power = 90%, α = 0.05, one tail, correlational cross-sectional study) indicated that 556 participants were required to detect an effect size of 0.25. The sampling lasted from May 2021 to August 2021.

### Measurements

Data were collected through demographic characteristics questionnaire, functional communicative critical health literacy (FCCHL), and treatment adherence questionnaire.

#### Demographic characteristics questionnaire

Demographic information included age, sex, job, income, smoking history, marital status, educational level, insurance, body mass index (BMI), HbA1c, family history of type 2 diabetes, duration of diabetes, type of medication, number of medications, and comorbidity related to type 2 diabetes.

#### Treatment adherence questionnaire

This questionnaire developed by Modanloo evaluates treatment adherence for chronic diseases (24). This Persian-language questionnaire consists of forty items with seven subscales based on a six-point Likert scale rating from always (five) to never (zero). Its subscales are interest in treatment (nine questions), willingness to participate in treatment (seven questions), ability to adapt (seven questions), integration of treatment with life (five questions), adherence to treatment (four questions), commitment to treatment (five questions) and management in the implementation of treatment (three questions). The minimum and maximum scores of interest in treatment, willingness to participate in treatment, ability to adapt, integration of treatment with life, adherence to treatment, commitment to treatment, and management in the implementation of the treatment were 0–45, 0–35, 0–25, 0–20, 0–25, and 0–15, respectively. According to the questionnaire developer, the initial scores are converted into scores between 0–100; a score of 75%−100% means very good treatment adherence, a score of 50%−74% means good treatment adherence, a 26%−49% score means average treatment adherence, and a score of 0–25% means a poor treatment adherence (24).

A few items were scored in reverse (33, 34–35, 37–39 and 40). Thus, it is possible to calculate the maximum and minimum scores for each subscale. The scoring is positive, meaning that the greater the total score or the score of each subscale, the greater the treatment adherence. This questionnaire also assesses diligence in the treatment process, willingness to engage in treatment, adaptability, integration of treatment into daily life, adherence to treatment, commitment to treatment, and hesitation to seek treatment (25). In Modanloo's study, the average content validity index (CVI) of the questionnaire was 0.914. The internal consistency of the questionnaire was determined by calculating Cronbach's alpha (α = 0.921), and the simple correlation of the questionnaire was *r* = 0.875.

The treatment adherence questionnaire has been used in various stages of clinical treatment and research in Iran. The reliability was determined using the Interclass Correlation Coefficient (ICC) index that was 0.921 for the entire questionnaire with a confidence interval of (0.94–0.9) (26).

#### Functional communicative critical health literacy

This questionnaire, which was validated in the study of Reisi et al. (27), assesses the level of health literacy of patients with diabetes. Exploratory factor analysis (EFA) identified three main factors with 27.07, 22.46, and 16.23% of extracted variance, respectively. Confirmatory factor analysis (CFA) completely supported the three-factor model of the health literacy (HL) scales. Internal consistency was approved for the total scale (α = 0.82) and for the functional, communicative, and critical subscales (α = 0.91, 0.80, and 0.76, respectively). Convergent validity analysis indicated a significant positive correlation (*r* = 0.45; *P* < 0.01) between the scores in the functional HL scale and the Iranian version of the Short Test of Health Literacy in Adults (S-TOFHLA). The questionnaire has 14 items in three sections: functional (five items about “reading instructions or leaflets from hospitals/pharmacies”), communicative (five items about “since being diagnosed with diabetes”), and critical (four items about “since being diagnosed with diabetes”). The answers to the questions were evaluated using a four-point Likert scale ranging from never to almost always. Each answer choice receives a score ranging from four to one (inverse). To calculate the health literacy score, all the scores of the items are added together and divided by whole questions. Scores were recoded for functional HL, and mean scores were calculated for each scale ranging from one (low HL) to four (high HL), with higher scores indicating higher levels of HL. In contrast to most HL screening tools, no cut-off point was available (28).

### Data collection and statistical analysis

In order to collect data, we first visited Diabetes Clinics in Kerman (Erfan Salamat, Bahonar, and Shafa) and received the necessary ethical permission. We introduced ourselves and explained briefly about the research, distributed the questionnaires among patients, who met the inclusion criteria, and reminded them that they could withdraw from the study at any time. Some patients were illiterate or too old to fill in the questionnaires, so we read questions one by one and the patients answered them. We used a similar tone and pitch for all patients and explained questions that needed further clarification.

SPSS18 was used to analyze data. The questionnaires were coded after data collection. Then, the data were analyzed using descriptive and inferential statistics. Descriptive tables, mean and standard deviation were calculated for each of the scales. Pearson correlation coefficient was used to check the correlation between quantitative variables. We used independent-*t* test and analysis of variance (ANOVA) to check the treatment adherence and health literacy scores according to study qualitative variables. Multivariable regression analysis was used to determine the predictors of treatment adherence. The multivariable normality distribution was examined according to the Mahalanobis criterion. Nineteen outliers were identified and removed from the analysis, so the regression analysis was performed on 685 data. Variance inflation factor (VIF), Tolerance (to check for multicollinearity), and Durbin-Watson test (to check the independence of measurement errors) were controlled all of which were acceptable. Linearity of residuals, independence of residuals, normal distribution of residuals, and equal variance of residuals were acceptable. Significance level was considered <0.05.

### Ethical considerations

The Ethics Committee of Tehran University of Medical Sciences approved this research with the code of Ethics No. IR.TUMS.SPH.REC.1399.250 and IR.TUMS.SPH.REC.1400.218. After receiving the necessary permission from officials of university and diabetes centers and obtaining written consent from each patient, we assured the research participants that their participation was voluntary and that non-participation in the study would have no impact on the delivery of healthcare services. The participants were also provided with the information and explanations regarding the research and questionnaire.

## Results

The study results showed a significant relationship between age (*P* = 0.03), body mass index (*P* = 0.005), HbA1c level (*P* < 0.001), and treatment adherence. We found a significant relationship between age (*P* < 0.001), HbA1c (*P* = 0.001), and health literacy (Table 1).

**Table 1:** Mean and standard deviation of quantitative demographic and characteristics and their relationships with treatment adherence and health literacy among study participants.

**Variable**	**Mean**	**SD**	**Treatment adherence**		**Health literacy**	
			***R***	***P*-value**	***r***	***P*-value**
Treatment adherence	52.64	4.12	–	–	0.54	**< 0.001**
Health literacy	2.20	0.49	0.54	**< 0.001**	–	–
Age (years)	62.04	9.68	−0.08	**0.03**	−0.25	**< 0.001**
BMI (kg/m^2^)	22.48	3.97	0.11	**0.005**	0.04	0.30
Duration of diabetes (years)	14.36	8.04	0.04	0.23	−0.02	0.57
Course of treatment (years)	14.04	7.91	0.04	0.35	−0.02	0.68
HBA1c level (%)	9.77	0.56	−0.29	**< 0.001**	−0.13	**0.001**

r, Pearson Correlation Coefficient. Bold values indicate the significance of that variable (*P* < 0.5).

According to Table 2, the majority of participants were female, married, unemployed, and had diploma and insufficient income. Most of the participants were under the coverage of social security insurance and had no history of smoking. According to the study results, the majority of patients used more than two medications, had a family history of diabetes, and received both oral medications and insulin therapy. We indicated a significant relationship between educational level, job, income, type of insurance, smoking, number of medications, family history of diabetes, and adherence to treatment. Table 2 indicated a significant relationship between sex, educational level, occupation, family income, type of insurance, number of medications, family history of diabetes, and health literacy.

**Table 2:** Mean and standard deviation of demographic and characteristics and their relationships with adherence to treatment and health literacy among study participants.

**Variable**	**Frequency**	**Percent**	**Treatment adherence**		**Statistical test (*P*-value)**	**Health literacy**		**Statistical test (*P*-value)**
			**Mean**	**SD**		**Mean**	**SD**	
**Sex (*****n*** **=** **704)**							
Female	520	73.9	52.30	4.03	*t* = −1.68 (0.09)	2.15	0.48	*t* = −5.03 **(< 0.001)**
Male	184	26.1	52.89	4.33		2.36	0.48	
**Marital status (*****n*** **=** **702)**							
Married	688	98.0	52.44	4.07	*t* = −0.84 (0.4)	2.20	0.48	*t* = −1.75 (0.08)
Single	14	2.0	53.38	6.07		2.43	0.65	
**Educational level (*****n*** **=** **704)**							
Uneducated	159	22.6	50.41	3.03	*F* = 37.70 **(< 0.001)**	1.70	0.22	*F* = 301.43 **(< 0.001)**
Primary	176	25.0	51.46	3.48		1.98	0.26	
Middle	110	15.5	52.49	3.59		2.24	0.30	
Diploma	180	25.6	53.64	4.25		2.56	0.32	
> Diploma	79	11.2	56.05	4.54		2.86	0.41	
**Job (*****n*** **=** **704)**							
Unemployed	439	62.4	51.87	3.79	*F* = 16.10 **(< 0.001)**	2.06	0.43	*F* = 59.52 **(< 0.001)**
Employed/Self-employed	86	12.2	52.44	4.49		2.37	0.52	
Retired	179	25.4	53.90	4.36		2.47	0.46	
**Family income (*****n*** **=** **698)**							
Sufficient	26	3.7	57.25	5.47	*F* = 33.89 **(< 0.001)**	2.76	0.48	*F* = 29.75 **(< 0.001)**
Relatively sufficient	313	44.8	53.16	4.11		2.28	0.49	
Insufficient	359	51.5	51.54	3.66		2.11	0.45	
**Insurance type (*****n*** **=** **703)**							
Social security	310	44.1	52.09	3.85	5.34 **(< 0.001)**	2.14	0.44	13.80 **(< 0.001)**
Armed forces	34	4.8	54.32	4.44		2.38	0.45	
Iranian	233	33.2	53.04	4.20		2.36	0.52	
Rural	35	5.0	50.69	3.44		1.91	0.44	
Others	91	12.9	52.21	4.49		2.07	0.45	
**Smoking (*****n*** **=** **703)**							
Yes	94	13.4	51.57	3.60	*t* = −2.25 (**0.02**)	2.16	0.45	*t* = −0.85 (0.40)
No	609	86.6	52.59	4.18		2.21	0.49	
**Number of medications used (*****n*** **=** **698)**							
One	12	1.7	56.54	5.93	*F* = 6.90 **(0.001)**	2.53	0.71	*F* = 10.68 **(< 0.001)**
Two	115	16.5	52.85	52.85		2.36	0.44	
More than two	571	81.8	52.30	4.06		2.17	0.48	
**History of family diabetes (*****n*** **=** **704)**							
Yes	489	69.5	52.74	4.30	*t* = 2.73 **(0.007)**	2.25	0.49	*t* = 4.02 **(< 0.001)**
No	215	30.5	51.82	3.58		2.09	0.46	
**Type of medication used (*****n*** **=** **704)**							
Oral	183	26.0	52.35	4.3	*F* = 0.20 (0.82)	2.22	0.52	*F* = 0.20 (0.82)
Insulin therapy	130	18.5	52.35	3.72		2.20	0.44	
Oral and insulin therapy	391	55.5	52.55	4.16		2.19	0.48	

t, independent t-test; F, analysis of variance; n, number of available data. Bold values indicate the significance of that variable (*P* < 0.05).

Due to the normal distribution of variables, we used independent t-test to compare the two groups, and analysis of variance to compare more than the two groups.

The mean score of treatment adherence in patients with type 2 diabetes was 52.64 ± 4.12. Hesitation to seek treatment (3.0 ± 0.5) and adaptability (2.29 ± 0.26) received the highest and lowest mean scores, respectively (Table 3). According to the questionnaire scoring, 18% of the samples (*n* = 127) had moderate adherence, while 82% (*n* = 577) had good adherence. The mean and standard deviation of health literacy was 2.20 ± 0.49, meaning that the patients had moderate health literacy; the communicative dimension (2.56 ± 0.47) and the critical dimension (1.48 ± 0.50) received the highest and lowest means, respectively.

**Table 3:** Comparison of the relationship between health literacy and treatment adherence among study participants.

**Variable**		**Mean**	**SD**	**Health literacy (Pearson correlation coefficient)**			
				**Functional**	**Communicative**	**Critical**	**Total score**
Treatment adherence	Due diligence in the treatment process	2.58	0.26	0.42	0.34	0.45	0.51
	Willingness to engage in treatment	2.68	0.14	0.28	0.22	0.34	0.35
	Adaptability	2.29	0.26	0.47	0.22	0.37	0.50
	Integration of treatment into daily life	2.83	0.14	0.30	0.18	0.40	0.36
	Adherence to treatment	2.57	0.25	0.37	0.18	0.34	0.40
	Commitment to treatment	2.70	0.44	0.36	0.21	0.35	0.40
	Hesitation to seek treatment	3.0	0.5	0.15	0.23	0.34	0.24
	Total score	52.64	4.12	0.45	0.29	0.45	0.54

In all cases *P* < 0.001.

The results indicated a direct and significant relationship between health literacy, all its dimensions, treatment adherence, and all its dimensions (*P* < 0.001). According to the results, the higher the health literacy of individuals, the higher the score of treatment adherence and vice versa (Table 3).

We used univariate and multivariate linear regression (stepwise method) to assess the predictors of adherence to treatment among patients with type 2 diabetes; Adherence to treatment was a dependent variable, while health literacy, age, BMI, HbA1c, level of education, job, family income, type of insurance, smoking, number of medications used, and family history of diabetes were independent variables. The results showed that health literacy, HbA1c, and income affected 35% of the variances in treatment adherence. As patients' health literacy increased, their adherence scores increased; an increase in their HbA1c level decreased their adherence score by 1.84. We found 2.54-point and 0.76-point increases in the treatment adherence scores of those with sufficient and relatively sufficient incomes (Table 4).

**Table 4:** Regression analysis of predictors for treatment adherence among patients with type 2 diabetes.

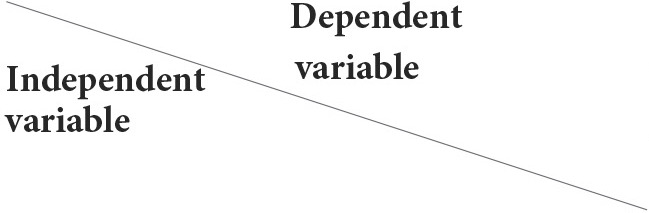	**Treatment adherence**							
	**Uni variable linear regression**				**Multi variable linear regression**			
	***b***	**SE**	**β**	**CI**	***b***	**SE**	**β**	**CI**
**Health literacy**	4.56	0.27	0.54	4.04 to 5.09	3.64	0.27	0.46	3.12 to 4.17*
**Age**	−0.03	0.02	−0.08	−0.07 to −0.003	–	–	–	–
**BMI**	0.11	0.04	0.11	0.03 to 0.19	–	–	–	–
**HbA1c**	−2.45	0.33	−0.29	−3.1 to −1.80	−1.84	0.28	−0.21	−2.39 to −1.28*
**Level of education**					
Uneducated	Ref	Ref	Ref	Ref	–	–	–	–
Primary	1.05	0.41	0.11	0.25 to 1.86	–	–	–	–
Middle	2.07	0.46	0.18	1.16 to 2.98	–	–	–	–
Diploma	3.23	0.41	0.34	2.43 to 4.03	–	–	–	–
**>** Diploma	5.64	0.52	0.43	4.63 to 6.65	–	–	–	–
**Job**					
Unemployed	Ref	Ref	Ref	Ref	–	–	–	–
Employed/ Self-employed	0.57	0.48	0.04	−0.36 to 1.50	–	–	–	–
Retired	2.03	0.36	0.22	1.33 to 2.73	–	–	–	–
**Family income**					
Insufficient	Ref	Ref	Ref	Ref	Ref	Ref	Ref	Ref
Relatively sufficient	1.65	0.30	0.20	1.05 to 2.24	0.78	0.25	0.10	0.29 to 1.27**
Sufficient	5.74	0.80	0.26	4.17 to 7.30	2.54	0.68	0.13	1.20 to 3.88*
**Insurance type**					
Social security	Ref	Ref	Ref	Ref	–	–	–	–
Armed forces	2.23	0.73	0.12	0.79 to 3.67	–	–	–	–
Iranian	0.94	0.35	0.11	0.25 to 1.63	–	–	–	–
Rural	−1.41	0.72	−0.07	−2.83 to 0.02	–	–	–	–
Others	0.12	0.48	0.01	−0.83 to 1.07	–	–	–	–
**Smoking**					
(No = 0, Yes = 1)	−1.03	0.46	−0.08	−1.92 to −0.13	–	–	–	–
**Number of medications used**					
One	Ref	Ref	Ref	Ref				
Two	−2.01	1.04	−0.18	−4.05 to 0.03	–	–	–	–
More than two	−2.56	0.98	−0.24	−4.48 to −0.63	–	–	–	–
**History of family diabetes**					
(No = 0, Yes = 1)	0.91	0.34	0.10	0.26 to 1.57	–	–	–	–

b, unstandardized coefficients; β, standardized coefficients; SE, standard error; CI, confidence interval.

*P < 0.001.

**P = 0.002.

R = 0.59, Adjusted R^2^ = 0.35, F = 82.21, P < 0.001.

## Discussion

This study aimed to determine the factors associated with treatment adherence in patients with type 2 diabetes in Iran. According to the study results, health literacy, HbA1c, and income affected treatment adherence and the higher the health literacy and income, the higher the treatment adherence. As patients' HbA1c reduced, their treatment adherence increased. Consistent with the study results, Hossain et al. (22) in Pakistan indicated that people with adequate health literacy remembered to take their medications more than those with poor health literacy, and they did not change the dose of their medication without medical advice. Olaloronpu et al. (29), Fan et al. (30), and Bauer et al. (31) supported the present study and found a positive and significant correlation between health literacy and treatment adherence. They suggested that healthcare providers should address health literacy to improve medication adherence and patient outcomes.

Nelson et al. (32) in the USA considered age and health literacy as factors influencing treatment adherence. Younger age and limited health literacy prevented treatment adherence in patients with type 2 diabetes (32). Their results were consistent with ours in terms of health literacy but inconsistent in terms of age because their study type, method, and the instruments were different (32). Adherence to treatment differs from one person to another due to their different conditions and characteristics. It seems that older people are more adhered than younger people are due to the fear of disease complications; although, the compliance rate in some older people may be lower due to some factors, such as forgetfulness, multiple medications, loneliness, etc.

Goli Roshan et al. (7) in Iran showed a significant relationship between health literacy and adherence to treatment. According to regression analysis, the higher the health literacy, the higher the rate of treatment adherence (7). Mehrtak et al. (33) in Iran reported the effect of health literacy on adherence to medication, nutrition, and physical activity of patients with type 2 diabetes. These results were in line with the results of the present study. Tahery et al. (34) in Iran indicated a positive relationship between health literacy, self-care, and self-efficacy. Self-care includes adhering to a healthy diet and physical activity, taking medication and foot care, and monitoring blood glucose (35); the results of this study were in line with the results of the present study. Kooshyar et al. (8) in Iran confirmed our results and emphasized that people with adequate health literacy had higher adherence to medication, diet, and physical activity and their HbA1c level was significantly low. Mosher et al. (36) in the USA reported a significant relationship between health literacy and knowledge, of medications, but no statistically significant relationship between health literacy and medication adherence. Mosher used rapid estimate of adult literacy in medicine (REALM) to measure health literacy of the research units. This instrument does not measure different aspects and skills of health literacy, but we used FCCHL that was more comprehensive than the rapid estimate of adult literacy in medicine; this difference might explain the various results. Huang et al. (37) in the USA rejected our results and believed that health literacy had no association with medication adherence or blood glucose control in patients with type 2 diabetes. Different results of the two studies can be due to different sample size (we used a larger sample size), data collection tools, and cultural differences.

Adherence to treatment is the most appropriate strategy for controlling type 2 diabetes, but insufficient health literacy is an important obstacle to patients' adherence to treatment. Healthcare officials and policy makers must pay more attention to health literacy in health promotion programs. Simplifying information and using memorable and understandable educational materials can help increase the level of health literacy; health professionals should provide appropriate training programs to improve the level of health literacy in patients with type 2 diabetes (33).

Gholamaliei et al. (9) in Iran found a significant relationship between age, level of education, healthcare cost, healthcare team and system, factors related to treatment and disease, beliefs related to disease, self-efficacy, concerns related to medication use, and adherence to medication. Tol et al. (38) in Iran found a relationship between marital status, sex, educational level, and treatment adherence; as individuals got older, their treatment adherence increased. Khanjani et al. (5) in Iran found a statistically significant relationship between sex, marital status, educational level, respectable behaviors of physician, respect for patient privacy, physician's skill and satisfaction, history of other diseases, history of hypertension, and medication adherence; their results were not consistent with our results due to cultural differences.

Older people tend to perceive the risk of diabetes more than younger ones, so they adhere to treatment better, but older people in our study had lower treatment compliance due to problems, such as forgetfulness, loneliness, multiple medications, fear of insulin injections, and some misconceptions in the field of medication use. People with higher education levels are aware of the complications of not taking medication, not following the diet, and not receiving medical instructions, which explain the relationship between education and adherence to treatment.

The positive relationship between the patient and the caregiver is one of the most important determinants of adherence to the optimal treatment, compassionate and empathetic caregiving increases patients' values, preferences, participations, decision-making independences, and adherence to treatment (5).

The studies conducted in America, Iran and Turkey supported our results, suggesting that having a higher income level was effective in the treatment adherence (39–41); people with a higher income level benefit from the services of the public and private sectors, more expensive drugs, and services at home. Those with insufficient incomes try to use governmental services less and ignore their treatment status due to the cost of food and accommodation (42, 43).

Studies conducted on patients with diabetes in the United Arab Emirates, Iran, North Carolina, the United States of America, and Southern California supported our results, suggesting an association between improved adherence and lower HbA1c (44–48). People with diabetes must control their blood sugar; people with higher treatment adherence regularly check their blood sugar, so their HbA1c levels are always good.

Tanharo et al. (49) in Iran indicated a relationship between diabetic foot, family history of diabetes, heart and kidney diseases, and treatment adherence; patients with diabetic foot ulcers or a family history of diabetes were more likely to seek treatment adherence because they were more aware of diabetes and its complications. Age, sex, economic status, smoking, and duration of diabetes had a reverse correlation with treatment adherence (49). Their results contradicted our results. In addition, patients with a history of heart and kidney diseases were more adhered because they were at greater risk, were more involved in the course of treatment, were aware of the factors affecting the process of diseases, and had more contacts with healthcare team; older people are at higher risk of developing diabetes complications. To treat diabetes and prevent its complications, the patient will need to adhere to treatment more. Tanharo found that patients' treatment non-adherence was due to an increase in the duration of diabetes.

Different results suggest the effect of many factors on the treatment adherence. We recommend further studies in specific geographical areas with different cultures. This study addressed some important knowledge gaps and examined a large number of patients with type 2 diabetes in Kerman city, so we can generalize the study results to other patients with diabetes in Kerman province; healthcare workers can use these results to identify factors associated with poor treatment adherence in patients with type 2 diabetes. They should design and implement supportive interventions for these patients using the study results.

This study coincided with the prevalence of COVID-19, so sampling was difficult due to quarantine. We were unable to do sampling virtually because getting the contact numbers of all patients was difficult, filling in the questionnaire virtually took time, and using the electronic questionnaire was difficult due to old age or illiteracy of some patients. Some elderly patients were impatient to answer the questions, so we tried to explain the research goals and attract their attention by improving conditions, such as sitting in a quiet place and providing proper air conditioning. We used self-reported data, so they might not reflect the actual performance of individuals. Our cross-sectional study was unable to understand the cause and effect relationship between the variables. Some patients ignored their usual check-ups during the outbreak of COVID-19, so bias occurred during the sampling process and we had no integrated and correct access to all patients. Generalization of these results to other parts of Iran is impossible; we require a study with a larger sample size.

## Conclusion

Our results suggested several factors affecting diabetes treatment adherence; health care professionals should focus on and minimize these factors to improve adherence in patients with type 2 diabetes. We indicated that interventions were necessary to reduce non-adherence in patients with type 2 diabetes; we can improve patient health by addressing treatment non-adherence among patients with type 2 diabetes with limited health literacy.

## Data availability statement

The raw data supporting the conclusions of this article will be made available by the authors, without undue reservation.

## Ethics statement

This study is part of the Ph.D. dissertation on health education and health promotion and research project (No. 55387). The dissertation (IR.TUMS.SPH.REC.1399.250) and research project (IR.TUMS.SPH.REC.1400.218) were approved by Tehran University of Medical Sciences. The patients/participants provided their written informed consent to participate in this study.

## Author contributions

NP collected the data. NP, RS, AT, and ES wrote the article. MY analyzed the data. MS provided advice to other authors on how to better communicate with patients. All authors read the article and reviewed it. All authors approved the final version of the manuscript.

## Conflict of interest

The authors declare that the research was conducted in the absence of any commercial or financial relationships that could be construed as a potential conflict of interest.

## Publisher's note

All claims expressed in this article are solely those of the authors and do not necessarily represent those of their affiliated organizations, or those of the publisher, the editors and the reviewers. Any product that may be evaluated in this article, or claim that may be made by its manufacturer, is not guaranteed or endorsed by the publisher.
